# Dietary utilization of mealworm frass in rabbit feeding regimes and its effect on growth, carcass characteristics, and meat quality

**DOI:** 10.3389/fvets.2023.1069447

**Published:** 2023-02-07

**Authors:** Mohamed A. Radwan, Aristide Maggiolino, Hanan A. M. Hassanien, Pasquale D. Palo, Nabila E. M. El-Kassas, Hassan S. Abbas, Abdelfattah Z. M. Salem

**Affiliations:** ^1^Department of Animal Production, Faculty of Agriculture, Cairo University, Giza, Egypt; ^2^Department of Veterinary Medicine, University of Bari A. Moro, Valenzano, Italy; ^3^Animal Production Research Institute, Agricultural Research Center, Giza, Egypt; ^4^Facultad de Medicina Veterinaria y Zootecnia, Universidad Autónoma del Estado de México, Toluca, Mexico

**Keywords:** growth performance, mealworm (*Tenebrio molitor*) frass, fatty acids, rabbit, meat quality

## Abstract

This study aimed to shed light on the use of mealworm (*Tenebrio molitor*) frass (TMF) in rabbit diets and its effects on growth performance, blood profiles, rabbit meat quality, and fatty acid profiles. A total of 48 Gabali rabbits were divided equally and randomly into four groups to be fed one of four dietary treatments: a control (T0) group and three TMF groups, which included TMF meal at 1 (T1), 2 (T2), and 3 (T3) %, respectively. The rabbits were fed on isonitrogenous and isoenergetic diets for 11 weeks, and the growth performance data were recorded. Six rabbits per group were slaughtered at the end of the 11th week, then the pharmacochemical parameters and carcass traits were measured, and meat quality and fatty acid profiles were analyzed. The results indicated that the growth performance of different groups was similar (*P* > 0.05). The levels of globulin, glucose, and alanine transaminase (ALT) were lower in the mealworm frass groups compared with the control group. Carcass traits were not affected by experimental regimes. Fat was higher in the TMF treatment groups, while moisture was lower compared with the control group. The shear force had a lower value in the treatment groups T3 and T2 compared with the control group. The highest values of redness and chroma (color parameter) of rabbit meat were observed in the treatment groups vs. the control group. Moreover, the proportion of total saturated fatty acids in the meat of rabbits that were fed on the T2 and T3 diets was higher compared to those fed on the control (i.e., T0) and T1 diets. Furthermore, the proportion of monounsaturated fatty acid was higher in the T2, T3, and T1 groups vs. T0 rabbits, whereas the PUFA proportions were lower. It could be concluded that frass has great potential to be used as a partial substitute for rabbit diets.

## Introduction

Sustainable farming practices are necessary to enhance food production (1), to cope with the increases in the human population, and to address the increasing need in recent years for alternative sources of protein for both humans and livestock. Insect production is projected to significantly grow in the short term due to its zero-waste sustainability (2).

Insects are paramount for ecosystems throughout the world and offer countless benefits for humans and livestock due to their rapid reproduction rates, recycling of organic matter (waste), and insect protein having high nutritive value (3). Insects or insect byproducts like frass are counted as useful feed ingredients for aquaculture, poultry, and rabbits, but also have the possibility to enhance livestock health and immune systems which may result in a decrease in antibiotic use. Studies have reported that insects could be utilized as a valuable nutrient instead of expensive feedstuff ingredients which could lead to the evolution of the agricultural economy (4–7). Mealworm is one of the most attractive insect species used as an alternative and sustainable feed source (8). Frass is a term referring to insect larvae excreta, which includes larval excrement, heterogeneous, exoskeleton sheds, and residual feed ingredients along with abundant nutrients, beneficial microbes, and chitin (4, 8–10). Frass is a good source of palmitic, myristic, and stearic acids which are defined as saturated fatty acids characterized by Benzertiha et al. (11) and Costa et al. (12). On the other hand, the total amount of monounsaturated fatty acids (MUFA) and polyunsaturated fatty acids (PUFA) are distinguished by a high content of oleic, linoleic, and linolenic acids (13, 14).

Rabbits produce around 1.8 million metric tons of meat per year. China provides 40% of the world's supply, followed by Italy, Spain, Egypt, and France (14.6, 3.8, 3.1, and 2.9%, respectively) (15). The addition of n-3 fatty acids to a rabbit's diet has been shown to improve the fatty acid composition of rabbit meat (16).

In this context, there is a shortage of information about the potential of using mealworm (*Tenebrio molitor)* frass as feed for livestock, particularly mammals. The main objective of this study is to explore the potential of mealworm frass as a feed ingredient and its effect on growth, carcass characteristics, meat quality, and the fatty-acid profile of rabbit meat.

## Materials and methods

### Experimental location

This study was conducted at El-Gharbia Governorate, El-Gemmaiza Station, which belongs to the Animal Production Research Institute (APRI), Agriculture Research Center (ARC), Ministry of Agriculture, Egypt. The chemical analysis was conducted in the laboratories of APRI, ARC, and Cairo University Research Park, Egypt (CURP).

### Frass

TMF was provided in the form of powder from the “Abou Abdo” farm located in Basos, Qalyubia Governorate, Egypt, which raises insects on a large scale. The mealworms were fed on agro-industrial byproduct or waste (wheat bran), in line with regulations for farm insect feeds. The frass was used without any chemical input after being sanitized at 70°C for 60 min.

### Experimental design

After being weaned at 6 weeks of age, forty-eight male Gabali rabbits were separated randomly into four groups according to weight (640± 40 g). A basal ration was prepared with ~17% crude protein (CP), which was supplemented with four levels of frass mealworm at 0 (T0), 1 (T1), 2 (T2), and 3 (T3) % of the feed instead of wheat bran and soybean meal. The rabbits were fed on isonitrogenous and isoenergetic diets for 11 weeks. Table 1 shows the four feeding diets as well as their chemical analysis. Table 2 contains the chemical analysis of mealworm frass that was performed according to AOAC (17). The Gabali rabbits were bred at the El-Gemmaiza breeding station in El-Gharbia Governorate, in affiliation with the Animal Production Research Institute (APRI), Agriculture Research Center (ARC), Ministry of Agriculture, Egypt. The rabbits were raised in cages with free access to water. Animals were weighed weekly for 11 weeks (the experimental period). These data were used to draw growth curves and compute body gain and average daily gain (ADG).

**Table 1:** Percentage composition determining the nutrient content of the different experimental diets.

**Item**	**T0**	**T1**	**T2**	**T3**
Clover hey	32	32	32	32
Yellow corn	8.45	8.49	8.63	8.63
Barley	22	22	22	22
Soybean meal 44%	17.23	17.19	17.15	17.05
Wheat bran	14	13	11.9	11
Mealworm frass (MF)	0	1	2	3
Molasses	3.5	3.5	3.5	3.5
Premix1 (minerals and vitamins)	0.5	0.5	0.5	0.5
DL-Methionine	0.12	0.12	0.12	0.12
Salt	0.5	0.5	0.5	0.5
Limestone	1.2	1.2	1.2	1.2
Di-Calcium phosphate	0.5	0.5	0.5	0.5
**Chemical analysis**				
C/P ratio	147.1	147.0	147.0	147.1
CP	17	17	17	17
CF	12.1	12.1	12.1	12.2
DE	2502.7	2502.3	2502.6	2501.6

Control (T0) = group fed on the basal diet, and T1, T2, and T3 = groups fed with dried mealworm frass levels of 1, 2, and 3%, respectively. c/p ratio, carbohydrate-protein ratio; CP, crude protein; CF, crude fiber; DE, digestible energy 1-provide per Kg of diet: 12000 IU Vit. A; 2.0 mg Vit.K; 310 mg Vit.E;2200 IU Vit.D3; 1.0 mg Vit.B1; 4.0 mg Vit.B2; 0.0010 mg Vit.B12; 6.7 mg; 1.5 mg Vit.B6; Vit. Pantothenic acid; 6.67 mg Vit. 1.67 mg Folic acid; B5; 1.07 mg Biotin; 400 mg Choline chloride; 10 mg Mn; 22.3 mg Zn; 25 mg Fe; 1.67 mg Cu; 0.033 mg Se;0.25 mg I and 133.4 mg Mg.l.

**Table 2:** The chemical composition of mealworm frass (on DM based).

**Item**	**DM**	**CP**	**CF**	**EE**	**Ash**	**OM**	**NFE**	**DE**
Mealworm frass (MF)	87.08	19.67	15.34	2.42	15.02	84.98	47.55	2445

DM, Dry mater; CP, crude protein; CF, crude fiber; EE, Ether extract; OM, organic matter; NFE, Nitrogen-free extracts were calculated as 100-% (moisture +CP +EE +CF +Ash); DE, digestible energy.

### Carcass merit, meat quality, and fatty acids profile

Six animals from each group were slaughtered in compliance with the requirements set by the Agricultural Research Center Institutional Animal Care, Care and Use Committee (ARC-IACUC). Carcass characteristics in terms of hot carcass weight, dressing percentage, and carcass dissection (three cuts: brisket, loin, and thigh) were conducted according to Abu Hafsa et al. (18). Chemical composition and physical traits as meat quality parameters were analyzed in the Meat Technology Lab at CURP. The thigh meat was used to determine chemical composition (moisture, protein, fat, and total collagen) by Food Scan™ meat analyzer (Foss Analytical A/S, Model 78810, Denmark). *Longissimus dorsi muscles* (LDM) of both sides were used to assess meat color, water holding capacity (WHC), cooking losses, and shear force. Assessment of meat color was conducted by a Chroma meter (Konica Minolta, model CR 410, Japan), measuring: lightness (L^*^, 0: dark to 100: light); the redness (a^*^) values, reddish (+value) to greenish (-value); and the yellowness (b^*^) values, yellowish (+value) to bluish (-value). Moreover, Chroma and hue were measured. The water holding capacity (WHC) percentage was measured according to Alagón et al. (19). The cooking loss percentage was measured according to Pascual et al. (20). The cooked samples were stored at 4–5°C for about 12 h and divided into 2–3 cubic pieces (1^*^1^*^ 2 cm). Each cube was tested twice using an Instron Universal Testing Machine (Model 2519-105, USA) with a V-shaped Warner-Bratzler shear blade, running at a crosshead speed of 200 mm/min.

The mixed meat from each group was prepared with [fatty acids (FA)] profile, 20 g of meat, and 100 ml of petroleum ether extract from the fat. Fat that was excreted was used for injection in HPLC.

### Blood sampling and analysis

Individual blood samples were collected during slaughter in heparinized test tubes (5 ml), centrifuged at 4000 rpm for 15 min to separate blood plasma, and then stored at −20°C ± 1 until the time of analysis to estimate blood parameters. The analysis was performed using a colorimetric technique according to the manufacturing guidelines of the kits (Diamond Diagnostics, Egypt), and included aminotransferase (AST), alanine aminotransferase (ALT), total protein, albumin, urea, glucose, cholesterol, and triglycerides.

### Statistical analyses

Data were analyzed using SAS^®^ On Demand for Academics with the general linear model (one-way). The statistical model: Y_ij_ = μ + F_i_ + eij was used, where Y_ij_ = the observation, μ = the overall mean, F_i_ = the effect of feeding system type (i= 1, 2, 3, and 4; where 1= control, 2= treatment 1, 3= treatment 2, and 4= treatment 3) and e_ij_ = random error. Tukey's test was applied to show the significant differences among the four groups (*P* < 0.05).

## Results

### The growth performance

**Figure 1:**
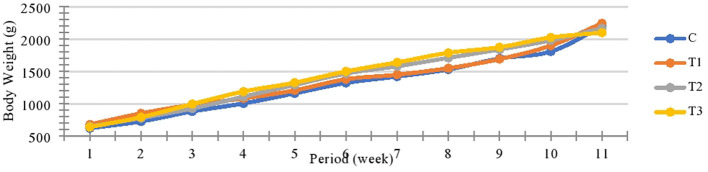
Growth curve pattern of different groups through the experimental period (C: control; T1: 1% mealworm frass in the diet; T2: 2% mealworm frass in diet; and T3: 3% mealworm frass in diet).

The growth curve of the different groups was taken in the same pattern. However, the initial weight, slaughter weight, total gain, and average daily gain (ADG) did not differ among the four groups (*P* < 0.05) (Table 3, Figure 1).

**Table 3:** The least-square means ± standard error of growth performance in the different groups studied.

**Item**	**T0**	**T1**	**T2**	**T3**
Initial weight, g	647 ± 22.8	680 ± 22.8	650 ± 22.0	640 ± 26.0
Slaughter weight, g	2200 ± 73.6	2115 ± 73.6	2166 ± 73.6	2100 ± 73.6
Total gain, g	1578 ± 62.0	1563 ± 62.0	1516 ± 59.8	1460 ± 70.7
Average daily gain, g	22.5 ± 1.0	22.3 ± 1.0	21.7 ± 1.0	20.9 ± 1.2

Control (T0) = group fed the basal diet, and T1, T2, and T3 = groups fed with dried mealworm frass levels of 1, 2, and 3%, respectively.

### Blood parameters

The total protein, albumin, blood urea, cholesterol, triglyceride, and AST was not significantly different among the four rabbit groups. However, globulin and glucose concentrations were lowest in the T3 group when compared with the T0 rabbits. The lowest ALT value was in the T3 and T2 vs. T0 rabbits. External environmental factors, including climate, management, and feeding, affected metabolism and influenced blood parameters. Protein fractions were estimated to assess the effect of mealworm frass on immunity function in rabbits, and increasing the level of mealworm frass slightly increased the concentration of total protein by up to 3% in diets (Table 4).

**Table 4:** The least-square means ± standard error of blood plasma parameters in the different studied groups.

**Item**	**T0**	**T1**	**T2**	**T3**
Total protein, g/dL	4.6 ± 0.1	4.6 ± 0.1	4.8 ± 0.1	4.7 ± 0.1
Albumin, g/dL	3.1 ± 0.2	3.0 ± 0.2	3.1 ± 0.2	3.5 ± 0.2
Globulin, g/dL	1.75 ± 0.1^a^	1.67 ± 0.1^ab^	1.72 ± 0.1 ^a^	1.42 ± 0.1^b^
A/G	2.06	1.87	1.82	2.92
Urea, mg/dL	3.59 ± 0.3	4.07 ± 0.3	4.02 ± 0.3	3.29 ±0.3
AST, IU/L	60.6 ± 4.1	58.9 ± 4.1	64.3 ± 4.1	66.8 ± 4.1
ALT, IU/L	86.8 ± 8.4^a^	71.8 ± 8.4^ab^	53.8 ± 8.4^b^	50.2 ± 8.4^b^
Glucose, mg/dL	106.8 ± 2.8^a^	106.3 ± 2.8^a^	103.0 ± 2.8^a^	90.7 ± 2.8^b^
Cholesterol, mg/dL	253.7 ± 17.7	250.8 ± 17.7	226.0 ± 17.7	206.8 ± 17.7
Triglyceride, mg/dL	210.4 ± 3.1	217.4 ± 3.1	214.40 ± 3.1	215.1 ± 3.1

Control (T0) = group fed the basal diet, and T1, T2, and T3 = groups fed with dried mealworm frass levels of 1, 2, and 3%, respectively. A/G, Albumin to globulin ratio; AST, aminotransferase; ALT, alanine aminotransferase. a and b least-square means in the same row with different superscripts were significantly different (*P* < 0.05).

### Carcass characteristics

Carcass weight, dressing percentage (DP), and dissection of the carcass (brisket, loin, and thigh percentage) were recorded as non-significant among the four groups, while the edible organs had the highest weight in T1 compared with T3 (*P* = 0.04). The boneless meat percentage was higher in the T3 group vs. other groups (*P* = 0.024) (Table 5).

**Table 5:** The least-square means ± standard error of carcass merits parameters in the different groups studied.

**Item**	**T0**	**T1**	**T2**	**T3**
HCW, g ^1^	1178 ± 58.8	1183 ± 58.8	1159 ± 58.8	1141 ± 58.8
Edible organs, g ^2^	84.8 ± 6.3^ab^	94.0 ± 6.3^a^	71.7 ± 6.3^b^	69.7 ± 6.3^b^
DP, % ^3^	55.1 ± 1.8	55.2 ± 1.8	55.8 ± 1.8	58.0 ± 1.8
Brisket, %^4^	39.7 ± 1.2	39.2 ± 1.2	39.1 ± 1.2	39.3 ± 1.2
Loin, % ^4^	22.0 ± 1.0	22.8 ± 1.0	22.0 ± 1.0	22.7 ± 1.0
Thigh, % ^4^	38.3 ± 0.4	38.0 ± 0.4	38.9 ± 0.4	38.0 ± 0.4
Bone less meat, % ^4^	78.4 ± 0.8^b^	76.7 ± 0.8^b^	78.8 ± 0.8^b^	82.0 ± 0.8^a^

Control (T0) = group fed the basal diet, and T1, T2, and T3 = groups fed with dried mealworm frass levels of 1, 2, and 3%, respectively. ^1^HCW: Hot carcass weight. ^2^Edible organs: liver+ heart+ kidney. ^3^DP: Dressing percentage. ^4^Calculated based on carcass weight. ^a, b^Least-square means in the same row with different superscripts were significantly different (*P* < 0.05).

### Meat quality

The moisture percentage of thigh meat had the highest value in the control group compared with other treatment groups, and the lowest recorded fat percentage of thigh meat was also in T0 vs. other treatments. Protein, collagen, water holding capacity (WHC), and cooking loss did not show any significant differences between groups. The lowest shear force value of rabbit meat was recognized in the T3 vs. other rabbit treatments. The color results and the L^*^, b^*^, and hue values were observed as non-significant among the four groups, while the lowest value of a^*^ and chroma was observed in the control group compared with the treatment groups (Table 6).

**Table 6:** The least-square means ± standard error of chemical composition and physical traits in the different groups studied.

**Item**	**T0**	**T1**	**T2**	**T3**
**Chemical composition, %** ^1^				
Moisture	75.6 ± 0.4^a^	75.1 ± 0.4^b^	75.0 ± 0.4^b^	74.8 ± 0.4^b^
Protein	22.5 ± 0.1	22.3 ± 0.1	22.3 ± 0.1	22.4 ± 0.1
Fat	1.9 ± 0.4^b^	2.4 ± 0.4^a^	2.7 ± 0.4^a^	2.6 ± 0.4^a^
Collagen	0.9 ± 0.1	0.9 ± 0.1	1.0 ± 0.1	0.9 ± 0.1
**Physical traits** ^2^				
WHC	71.7 ± 3.0	73.0 ± 3.0	71.9 ± 3.0	74.5 ± 3.0
Cooking loss, %	30.5 ± 1.0	29.1 ± 1.0	31.1 ± 1.0	30.3 ± 1.0
Shear force, (kgf/cm^3^)	3.0 ± 0.1^a^	3.0 ± 0.1^a^	2.7 ± 0.1^b^	2.6 ± 0.1^b^
**Color**				
L*	53.9 ± 0.9	54.1 ± 0.9	54.3 ± 0.9	52.6 ± 0.9
a*	14.0 ± 0.6^c^	15.7 ± 0.6^b^	17.1 ± 0.6^a^	15.7 ± 0.6^b^
b*	8.6 ± 0.6	9.0 ± 0.6	9.5 ± 0.6	8.2 ± 0.6
Chroma	16.8 ± 0.5^b^	18.6 ± 0.5^a^	19.4 ± 0.5^a^	18.2 ± 0.5^a^
Hue	31.2 ± 1.5	29.5 ± 1.5	29.1 ± 1.5	28.5 ± 1.5

Control (T0) = group fed the basal diet, and T1, T2, and T3 = groups fed with dried mealworm frass levels of 1, 2, and 3%, respectively. ^1^Applied to thigh muscle. ^2^Applied to loin muscle, WHC, water holding capacity, L^*^: lightness, a^*^: redness, b^*^: yellowness. ^a, b^Least- square means in the same row with different superscripts were significantly different (*P* < 0.05).

### Fatty acids

The percent of palmitic acid (C16:0) was higher in the muscles of the T2 and T3 groups (4.09 and 3.80 % of the total fatty acids) than in those of the control and T1, respectively. Furthermore, the difference in the percentage of total saturated fatty acids developed in the muscles of the rabbits fed the T2 and T3 diets compared to those fed the control and T1 diets was C15:0 and C16:0, respectively. The percentage of MUFA was also higher in the T2, T3, and T1 groups than in T0 rabbits, whereas the PUFA percentage was lower. Oleic acid (C18:1) was the main MUFA in the meat of the mealworm frass groups (26.8, 26.1, and 26.0% of the total FA for T2, T1, and T3, respectively) and MUFA rates were higher in mealworm frass groups than the control. The most abundant PUFA, linoleic acid (C18:2n-6), had a higher concentration of the total FA for T0 and T1 rabbits, at 22.8 and 21.1%, respectively. The meat of T0 and T3 rabbits had a higher proportion of C18:3 n3; however, Food and Agriculture Organization (FAO)/WHO committee, advised nutritional recommendations concer-ning the n-6/n-3 PUFA ratio, suggesting a value between 5:1 and 10:1, as reported in the International Society for the Study of Fatty Acids and Lipids (Table 7) (21).

**Table 7:** Least square means ± standard error of fatty acid profile in the meat of the different groups studied (g/100g of total acid methyl esters).

	**Mealworm frass (TMF)**			
**Fatty Acid**	**T0**	**T1**	**T2**	**T3**
C14:0	3.77	3.77	4.09	3.8
C15:0	0.18	0.76	0.85	0.77
C16:0	28.82	31.1	31.34	29.15
C17:0	1.01	0.76	0.9	1.05
C18:0	7.19	7.44	6.91	6.98
C20:0	0.51	0.2	0.15	1.6
C22:0	0.7	0.57	0.28	0.46
C24:0	0.41	0.2	0.16	0.63
Total saturated fatty acids (SFA)	41.4	43.28	42.93	42.62
C14:1	0.42	0.35	0.43	0.41
C16:1n-7	4.89	4.54	4.95	4.78
C17:1	0.7	0.33	0.48	0.74
C18:1n-9	25.02	26.07	26.85	26.00
C20:1	Nd	0.33	0.41	0.35
Total monounsaturated fatty acids (MUFA)	31.03	31.62	32.71	32.28
C18:2n-6	22.8	21.1	20.09	19.8
C18:3n-3(LNA)	2.36	2.23	2.12	3.49
C22:5n-3(DPA)	0.59	ND	ND	ND
Total polyunsaturated fatty acids (PUFA)	25.75	23.33	22.41	23.29
UFA	56.78	54.95	55.12	55.57
n-9	25.02	26.07	26.85	26.00
n-6	22.80	21.10	20.09	19.80
n-3	2.95	2.23	2.12	3.49
n-6/n-3 ratio	7.73	9.46	9.48	5.67
Unsaturated/Saturated	1.37	1.27	1.28	1.30

Control (T0) = group fed the basal diet, and T1, T2, and T3 = groups fed with dried mealworm frass levels of 1, 2, and 3%, respectively. C14:0 = Myristic acid; C15:0 = Pentadecanoic acid; C16:0 = Palmitic acid; C16:1 = Palmitoleic acid; C17:0 = Heptadecanoic acid; C18:0 = Stearic acid; C18:1n9c = Oleic acid; C18:2n6c = Linoleic acid; C18:3n3 = α-Linolenic acid; C20:0 = Arachidic acid; C20:1c = cis-11-eicosenoic acid; C20:2c = cis-11,14-eicosadienoic acid; C20:4n6 = Arachidonic acid; C20:5n3 = Eicosapentaenoic acid; C22:0 = Behenic acid; C24:0 = Lignoceric acid. ND: not detected. saturated fatty acids = SFA; monounsaturated fatty acids = MUFA; polyunsaturated fatty acids = PUFA; unsaturated fatty acids = UFA; n-6:(C18:2n6, C18:2n6, C18:3n6, C20:3n6, C20:4n6, C22:4n6); n-3:C18:3n3, C22:5n3.

## Discussion

Several studies have examined insects and their potential in the feeding system of livestock (22, 23). Mealworm in particular was investigated by Kowalska et al. (24), who found that mealworm larvae meal in rabbit feeding systems could be utilized with no adverse effect on growth traits, meat quality, or fatty acid profiles. Nevertheless, there is very little literature available regarding the possibility of using mealworm frass as an alternative protein source for rabbits. The experimental rations contained the same ingredients and were isoenergetic and isoproteic, so as to avert any possible discomposing effects of different energy levels due to lipid source substitution from the use of different ingredients. According to our results, growth performance was not affected by the usage of mealworm frass as part of the animal feeding system when compared with conventional feed. Gasco et al. (22) found that added insect fat in the feeding system of rabbits did not influence growth performance, and the same results were reported by Gessner et al. (25) who found that the growth performance of pigs was not influenced when they were fed on mealworm larvae as a source of protein, meaning there was no negative effect on metabolism. All carcass traits recorded values without statistical significance in the four groups with the exception of edible organs having a lower value in T2 and T3 vs. T1 rabbits. The significant differences in the edible organs among treatment groups could be correlated with carcass weight as mentioned by Elamin (26), who found a positive correlation between carcass weight and edible organs (liver and kidney). Carcass traits were not affected by the TMF added to diets. Similarly, these results agreed with the findings of (27, 28) that there was no effect of mealworm larvae (alternative source of protein) on the carcass traits of chicken. In contrast, Ballitoc and Sun (29) mentioned that when broiler chickens were fed TM (*Tenebrio molitor*) diets, it improved dressed carcass, slaughter, and eviscerated weights.

Blood parameters contribute to understanding the physiological status of livestock, which reflects the relationship between their diet and health status (30). Data from blood parameters in terms of total protein, glucose, urea, cholesterol, triglyceride, AST, and ALT levels were within the range of reference values reported for rabbits in previous studies (31, 32). All blood parameters had no significant differences among the four groups, except serum globulin, glucose, and ALT, whereas the blood globulin level in rabbits fed on mealworm frass was lower than in the control rabbits. In a recent study of *Tenebrio molitor* larvae meal used to feed broilers, results in the group fed insect meal showed lower albumin to globulin ratio even when no differences were found in globulin content. These results may be due to the properties of chitin contained in insect meal (33). Values of serum glucose were higher than that reported by other studies (34, 35) and lower than in others (36). However, raised glucose levels in rabbits can also be due to various factors such as stress, blood collection methods, transportation, unfamiliar noises, smell, chronic pain, poor environment, and housing conditions (32, 35). There was a link between blood glucose, food intake, signs of stress, and clinical disease severity. Rabbits with signs of stress had higher blood glucose levels than rabbits with no signs of stress, and rabbits that were completely anorexic had higher blood glucose levels than those who were eating normally or less feed intake.

Important indicators of liver functions are AST and ALT enzyme activities. According to Gasco et al. (22), rabbit diets including insect lipids did not influence liver enzymes. Levels are used to evaluate the effect of mealworm frass on fat metabolism AST. AST activity was near the levels reported in some previous studies (37) and lower than the levels observed in others (31, 32). However, the activity of ALT was lower than the values reported in some previous studies (32, 35). Blood urea nitrogen is positively correlated with protein in the diet. Urea is estimated to evaluate the effect of mealworm frass on kidney function because urea concentration is elevated when kidney function is disturbed due to exposure to toxic compounds. The values of the lipogram profile, including cholesterol, follow the same trend (38) according to results which show that reductions in cholesterol and triglycerides due to chitin could attract negatively charged bile acids and free fatty acids to its positive charge (39).

Rabbit thighs were used to evaluate the chemical composition according to the recommendation of Pla et al. (40). The fat percentage was higher in treatment groups, which received mealworm frass, than in the control group, while the moisture percentage followed the inverse trend; this indicates that using mealworm frass could enhance the meat flavor due to fat content (41). Meat tenderness (opposite to shear force value) was improved by using mealworm frass compared with the control group, which, along with meat fat, could be linked (42, 43) to the findings that adding mealworm to the diet of broiler chickens enhanced meat tenderness. The color of meat is considered an important factor that affects a consumer's purchasing decision. Redness and chroma were increased by using mealworm frass. This result indicated that the meat of rabbits fed on mealworm frass had a more reddish intensity, similar to the results reported by Zadeh et al. (44), where using mealworm meal up to 3% of dry matter increased the redness trait of Japanese quails.

The fat composition of rabbit meat is characterized by a significantly higher proportion of polyunsaturated fatty acids than found in other meats and low levels of saturated fatty acids (SFA) (16). The meat quality of rabbits may be influenced by several factors, especially lipid diet quantity and quality. The absorption of fat can be affected by the fat source. The composition of fatty acids is the main characteristic of rabbit meat due to differences in chain length, degree of saturation, and the degree of esterification of fatty acids (45). The lipid content and fatty acid composition of yellow mealworm (*Tenebrio molitor* L.) have been characterized. The predominant fatty acids in *Tenebrio molitor* L. fat were oleic (C18:1 c9), linoleic (C18:2 n6), and palmitic (C16:0) acids, and the total lipids represented 43% of the DM (46). Due to its high linoleic acid content, mealworm fat may be considered a source of n-6 PUFA (47). According to Nowakowski et al. (48), the health benefits of insects for both humans and other animals are due to high levels of omega-3 and−6 fatty acids, antioxidants, vitamins, fiber, minerals, and essential amino acids. Recent research in pigs indicated that fatty acids play a potential therapeutic role in enteritis and have an impact on the intestinal integrity of pigs (49). As a result of a higher amount of PUFA in the meat, lipid oxidation occurs and leads to the degradation of n-3 PUFA; in oxidative products, we can say that a meat's oxidative stability is worse when the PUFA content of meat increases (50). This means that a treatment diet with mealworm frass enhances the stability of oxidation of rabbit meat.

## Conclusion

Mealworm frass has the potential for use in the feeding system of rabbits (mammals) without unfavorable effects on growth performance and carcass traits, as well as improving meat quality parameters. Mealworm frass has health benefits due to its composition, although more research is needed on the bioactive components of insect frass.

## Data availability statement

The original contributions presented in the study are included in the article/supplementary material, further inquiries can be directed to the corresponding authors.

## Ethics statement

The animal study was reviewed and approved by the Animal Production Research Institute, Agricultural Research Center, Egypt Ethical Committee LACUC protocol number: ARC–AP-22-26.

## Author contributions

MR, HH, NE-K, and HA designed the experiment, carried out the research, and laboratory analysis. MR, HH, PP, AM, and AS conducted the data analysis, wrote the manuscript, and revised the manuscript. All authors contributed to the article and approved the submitted version.
